# Did the pandemic change lifestyle behaviours in Italy? An interrupted time series analysis on the four main NCDs behavioural risk factors from 2008 to 2023

**DOI:** 10.1186/s12889-025-22062-2

**Published:** 2025-02-27

**Authors:** Federica Asta, Valentina Minardi, Benedetta Contoli, Valentina Possenti, Virginia Casigliani, Maria Masocco

**Affiliations:** 1 National Centre for Disease prevention and health promotion, Istituto Superiore di Sanità, Rome, Italy; 2 Department of translational research and new technologies in Medicine and Surgery, University of Pisa, Pisa, Italy

**Keywords:** Interrupted time series, Surveillance systems, NCDs behavioural risk factors, Tobacco use, Harmful use of alcohol, Unhealthy diet, Physical inactivity, COVID-19, Public health strategies

## Abstract

**Background:**

The COVID-19 pandemic has had repercussions in several areas. The indirect effects of the pandemic on healthy living behaviours are multiple and complex to assess. The aim is to assess the impact of the outbreak of COVID-19 pandemic in Italy on the most relevant modifiable behaviours risks for non-communicable diseases (NCDs).

**Methods:**

PASSI 2008–2023 data referring to a sample of 18-69-year-olds residing in Italy was used to estimate the prevalences of smoking, alcohol, fruit and vegetable consumption, and physical inactivity lifestyle. For each of these risks was used an interrupted time series (ITS) study with Generalized Least Squares (GLS) model to assess trends before and after the pandemic’s outbreak in Italy (March 2020). The “intervention” period is postulated as March 2020, and the “post-intervention” period extends from April 2020 to December 2023. In these models, the hypothetical situation without “intervention” and with the trend remains unchanged is commonly known as the ‘counterfactual’ scenario. Through ITS model both “counterfactual data” and “factual data” were obtained.

**Results:**

From 2008 to 2023, 532,115 people were interviewed. Results showed, during “post-intervention” period, significant differences between factual and counterfactual prevalences started in 2022 and strengthened during 2023 and for all of the four behavioural indicators analysed: smoking, high-risk alcohol consumption, fruit and vegetable consumption (both all three of them worsening) and physical inactivity (which appears to decrease). Current smokers exhibited similar prevalences in March 2020 (24.5% vs. 24.3%), followed by a plateau throughout the entire ‘post-intervention’ period. By December 2023, however, the counterfactual prevalence was significantly lower than the observed rate (24.5% factual vs. 22.7% counterfactual). The initial improvement observed in the prevalence of high-risk drinkers in March 2020 (13% factual vs. 17% counterfactual) was followed by a rapid worsening and in December 2023 the prevalence was significantly higher than expected (19.7% vs. 16.9%). The fruit and vegetable consumption worsened and the factual prevalence of five-a-day in December 2023 was significantly lower than counterfactual (6.6% vs. 9.0%). Physical inactivity following an initial worsening in March 2020 (albeit not statistically significant), appeared to decrease in December 2023, with the factual prevalence lower than counterfactual (26.9% vs. 32.4%).

**Conclusions:**

Results found in this article showed at the end of studied period the achievement of a plateau for current smokers, a worsening in the high-risk alcohol consumption, in fruit and vegetable intakes and a reduction in physical inactivity. Understanding shifts in these lifestyle indicators is crucial for the proper design of interventions aimed at reducing the burden of NCDs.

**Supplementary Information:**

The online version contains supplementary material available at 10.1186/s12889-025-22062-2.

## Background

The COVID-19 pandemic affected everyone’s life: it caused about 7 million deaths [1] and led governments to adopt unprecedented measures to curb the spread of the virus, which had an important social and economic impact [2]. During the first phase of the pandemic, interventions, such as lockdowns, physical distancing and curfews, influenced people’s mental health, psychological well-being and habits [3]. According to the World Health Organization (WHO), most non-communicable diseases (NCDs), which represent the leading cause of mortality worldwide, are the result of preventable risk factors, in particular four lifestyle behaviours (tobacco use, physical inactivity, unhealthy diet, and the harmful alcohol consumption) that lead to four metabolic and physiological changes (raised blood pressure, overweight/obesity, raised blood glucose and raised cholesterol). Hence, 80% of premature heart disease, stroke, and diabetes can be prevented [4].

Healthy dietary habits, such as eating many fruits and vegetables and limiting high-calorie foods, are fundamental in preventing NCDs: current dietary recommendations indicate the intake of 5 servings of fruits and vegetables per day as the quantity associated with the lowest mortality and highest benefit for the health [5]. These habits seem to be affected by the pandemic, with a shift to higher consumption of snacks, as well as sweet and high-processed foods [6]. The increase in stress, anxiety, depression, and sleep disorders may have contributed to this change, being associated with an increase in food intake, especially less healthy. Besides, preventive measures to avoid physical contact adopted in many countries, may have negatively impacted eating habits, including among people with higher body mass index or with an eating disorder [7].

These measures, in particular lockdowns and curfews, also severely affected physical activity [8, 9]. It is well recognized that physical activity improves all-cause mortality, as well as cognitive health and sleep. According to a recent publication of the OECD, increasing physical activity to the minimum levels recommended by WHO would prevent 11.5 million new cases of NCDs by 2050, including 3.8 million cases of cardiovascular diseases, in the European Union (EU) [10]. The report also underlined the negative impact of the pandemic on EU citizens’ physical activity, with an increase in people who reported to have stopped being active in the last Eurobarometer survey.

Smoking and alcohol consumption constitute two well-known behavioural risk factors for many health conditions. The effects of the pandemic on smoking behaviours are uncertain: according to a meta-analysis reported data from 24 countries, during the pre-vaccination phase the overall prevalence of smokers decreased, and among people who smoked a similar proportion increased or decreased the tobacco, however with high heterogeneity [11, 12]. 

Harmful use of alcohol is accountable for 5% of the global burden of disease [13]. During the pandemic, alcohol consumption, and the frequency of use, including heavy episodic drinking, had different trends among the countries [14]. In Italy, according to a representative survey conducted among young adults during the lockdown in 2020, there was observed a reduction in overall alcohol use among people who drink moderately, while an increase in the prevalence of high-risk drinkers [15]. The indirect effects, particularly the long-term ones, of the pandemic on healthy lifestyle behaviours are multifaceted and complex to assess. Italy, one of the most affected countries in Europe, swiftly implemented significant restrictions, including lockdowns and physical distancing measures, during the initial phase of the pandemic.

Several studies, conducted in many countries included Italy, have investigated how healthy habits were modified during COVID-19 pandemic. However, most of them were ad hoc surveys focused on specific population subgroups using convenience samples, sometimes collected online, and typically involve short observation periods (one or two months) [6–9, 11, 12, 14]. Although these ad hoc surveys have provided interesting results in the immediate period and, included in important reviews, have contributed to synthesizing the available evidence, we believe it is really useful and important to have a comprehensive understanding of what has happened in the medium/long term regarding modifiable behavioural risks for NCDs through robust and representative samples of the general population.

This study aims to assess the impact of the COVID-19 pandemic outbreak on the four main behavioural risk factors within the Gaining Health framework [16]—tobacco use, harmful alcohol consumption, unhealthy diet and physical inactivity—among a representative sample of adult population residing in Italy, using a robust study design such as Interrupted Time Series (ITS). The analysis is based on monthly prevalence trends from 2008 to 2023, based on data of the Italian Behavioural Risk Factor Surveillance System PASSI (Progressi delle Aziende Sanitarie per la Salute in Italia) [17].

## Methods

### Data collection

PASSI is an ongoing cross-sectional Italian Surveillance System. It is based on the international framework of the US Centers for Disease Prevention and Control (CDC) [18], mandated by the Italian Ministry of Health and officially acknowledged in accordance with the Decree of the Prime Minister’s Office on Registries and Surveillances, 3 March 2017 [19]. The National Institute of Health in Italy, known as Istituto Superiore di Sanità – ISS, is responsible for coordinating this surveillance at national level.

Since 2008, PASSI collects data on health and behavioural risk factors among the adult population residing in Italy aged 18 to 69. The ongoing nature of PASSI entails the systematic collection of information from samples representative in terms of age and gender comprising approximately 33,000 individuals each year.

The Local Health Unit (LHU) in Italy organizes and provides most of health services and serves the primary unit for data collection in PASSI. Within each LHU, a monthly random sample is extracted from LHU’s enrolment list of residents, stratified by gender and age groups (18–34, 35–49, and 50–69) proportionally to their representation in the general population. The exclusion criteria are having the primary residence in another region, not having a valid telephone number, being currently hospitalized or in long-term care, nursing homes or prisons. Professionals with specialized training from the public health departments of LHUs conduct telephonic interviews employing a standardized questionnaire, pertaining to socio-demographic attributes, behaviour-associated risk factors, and physical and mental well-being. Data gathered in each LHU are then merged, weighted, and analysed to obtain regional and national estimates. PASSI samples are representative of the general adult population residing in Italy by age and sex. A detailed description of the validated survey design and random sampling procedures is available elsewhere [17, 20].

The continuous nature of PASSI data collection allows to estimate monthly prevalences of outcome of interest and evaluate change occurred also in a short time. In the present study, PASSI dataset referred to adults aged from 18 to 69 years and collected from January 2008 to December 2023 was used.

### Study outcomes and variables’ definition

Current smokers, high-risk drinkers, people who eat five or more fruit and vegetable portions and prevalence of physically inactive individuals, were identified from PASSI dataset, to assess the change in trends of the four over mentioned modifiable behaviours risks.

A current smoker is defined as a person who declared to have smoked at least 100 cigarettes (5 packs of 20) in his life and is currently a smoker, following the WHO definition [21].

High-risk drinkers are defined if respondents were at least in one of the following categories: heavy drinkers are men drinking more than 2 alcoholic units (AUs) per day on average or women drinking more than 1 AU; alcohol drinkers mainly outside of meals; binge drinkers (5 drinks or more in one occasion for men, and 4 for women) [22].

People eating at least five daily servings of fruits and vegetables (*five a day*) are identified through a single question that investigates habitual fruits and vegetables consumption. The surveillance does not collect any information on the intake of specific foods [23].

In line with 2020 WHO recommendations, physically inactive people are identified as those do not perform any vigorous or moderate-intensity aerobic physical activity during leisure time (such as running, bicycling, brisk walking, vacuuming) and do not perform regular jobs that requires considerable physical effort [24].

### Data analysis

For each of the four behavioural risk factors the monthly time series of weighted observed prevalences were calculated from January 2008 (2010 for high-risk alcohol consumption) to December 2023.

To evaluate the impact of the outbreak of COVID-19 on the prevalence trends of the four mentioned behavioural risk factors, we used an interrupted time series (ITS) study using Generalised Least Squares (GLS) model, accounting for autocorrelation.

In the ITS model the “intervention” period was considered March 2020, the month in which epidemiological emergency due to COVID-19 was declared in Italy, while “post-intervention” period was defined considering the months from April 2020 to December 2023.

ITS methodology is used to analyse, having a time series of outcome of interest, a trend that could be interrupted by a specific event at a known point in time (in this case the outbreak of the COVID-19 pandemic in Italy). In these types of models, the hypothetical situation in which the “intervention” did not occur and the trend remains unchanged is commonly known as the ‘counterfactual’ scenario. The counterfactual is the basis for assessing the impact of the “intervention” by examining any change that occurs in the post-intervention period. In this study, to create the counterfactual model, the time series of monthly prevalences truncated at the time point immediately before the intervention was created and the ‘predict’ command of the gls model was used [25]. Finally, we obtained “counterfactual data” and “factual data” both through ITS model. For unplanned events like the COVID-19 pandemic, determining the exact timing of the intervention can be challenging. Therefore, in our sensitivity analyses, we tested two different “intervention” periods first April 2020 and then May 2020 comparing any variations in the step change and the corresponding *p*-value [26].

In time series models the issue of autocorrelation, the tendency of outcomes in one month to be more similar to outcomes in adjacent months during the same season, needs to be addressed; GLS model allowed to add autoregressive integrated moving average (ARMA) matrix by specifying values for p (the autoregressive order) and q (the moving average order). It is important to choose the correct values of p and q, so the combination of values of p and q that gave the smallest value of the Akaike Information Criterion (AIC) for each model was considered.

All statistical analyses were conducted using the software R Studio [27].

## Results

A total of 95 out of 105 LHUs at national level participated in PASSI surveillance system (LHUs partecipation rate 90%). From 2008 to 2023, 532,115 people were interviewed, according to the American Association for Public Opinion Research standard definition (AAPOR) [28], the response rate varied over time from 83% in 2008 to 74% in 2023.

The mean age of respondents was 44 years and 49.0% were males. Most of the respondents had a high level of education (64.0%), almost half reported having economic difficulties (52.4%), and 4.3% of the sample was born foreign. The same prevalence of respondents lived in the North (38.5%) and in the South and Islands (38.8%).

Following the AIC criterion to select the optimal ARMA matrix in gls models, we used a value of p and q equal to one for all indicators to take into account the correlation issue.

**Fig. 1:**
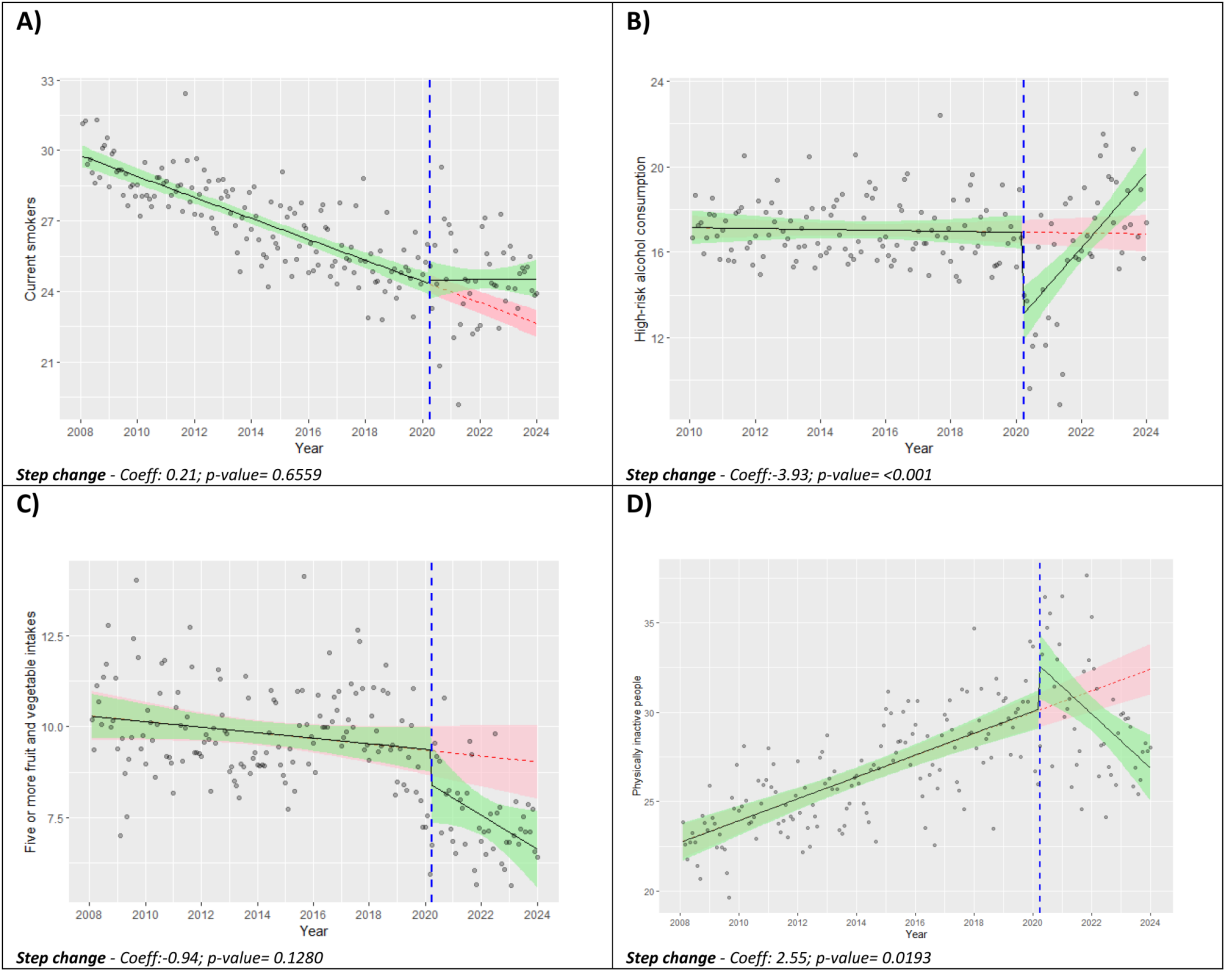
Interrupted time series models of monthly prevalence for: (**A**) current smokers*; (**B**) people at high-risk alcohol use; (**C**) Five or more fruit and vegetable intakes; (**D**) physically inactive people. Counterfactual data (red line) and factual data (green line) with relative 95%IC, and weighted observed data (grey dots). PASSI 2008–2023

The prevalence trends of the four main behavioural risk factors for the interrupted time-series analysis with their step changes and relative *p*-values are presented in Fig. 1.

For current smokers a plateau is observed, contrasting with the gradual deceleration of the downward trend seen in the counterfactual scenario (Fig. 1A). A sudden decrease followed by a rapid increase was noted in the prevalence of high-risk drinkers after the period in which epidemiological emergency due to COVID-19 was declared (Fig. 1B). An immediate worsening in time series was observed for people who eat five or more fruit and vegetable portions (Fig. 1C). A sudden increase followed by a rapid decrease was noted in the prevalence trend of physically inactive people (Fig. 1D).

The trends, immediately visible in the previous figure, were summarized in Table 1. It was chosen to present in the table only the counterfactual data and factual data for January 2008, March 2020 and December 2023.

**Table 1:** Changes in prevalence with CI95% for four main behavioural risk factors in the first and last months of the post-intervention period

Outcome		January 2008			March 2020			December 2023		
		Prevalence	CI 95%		Prevalence	CI 95%		Prevalence	CI 95%	
Current Smokers*	Factual	29.75	*29.30*	*30.20*	24.48	*23.69*	*25.27*	**24.54**	***23.75***	***25.33***
	Counterfactual	-	*-*	*-*	24.32	*23.94*	*24.70*	**22.65**	***22.08***	***23.21***
High-risk alcohol users ^$^	Factual	17.16	*16.37*	*17.95*	**13.16**	***11.91***	***14.41***	**19.70**	***18.44***	***20.95***
	Counterfactual	-	*-*	*-*	**16.95**	***16.40***	***17.49***	**16.87**	***16.01***	***17.73***
People who eat 5 or more fruit and vegetable portions	Factual	10.28	*9.67*	*10.89*	8.39	*7.35*	*9.42*	**6.64**	***5.58***	***7.69***
	Counterfactual	-	*-*	*-*	9.33	*8.64*	*10.01*	**9.03**	***8.02***	***10.04***
Physically inactive people	Factual	22.76	*21.70*	*23.82*	32.57	*30.75*	*34.38*	**26.89**	***25.06***	***28.72***
	Counterfactual	-	*-*	*-*	30.13	*29.17*	*31.09*	**32.39**	***30.97***	***33.81***

^*^ Only cigarette use

^$^ data available from 2010

The decision was made to incorporate the prevalence values for the phenomenon in Table 1, reflecting data from January 2008 (2010 for alcohol consumption), was made to provide an overview of the order of magnitude at the beginning of the observation period.

Table 1 illustrates that, during the “post-intervention” period, significant differences between factual and counterfactual prevalences are observed almost exclusively in December 2023. These differences apply to all four analysed risk factors: smoking, high-risk alcohol consumption, and fruit and vegetable intake each showing a worsening trend, while physical inactivity appears to decrease. Current smokers exhibited similar prevalences in March 2020 (24.5% vs. 24.3%), followed by a plateau throughout the entire ‘post-intervention’ period. By December 2023, however, the counterfactual prevalence was significantly lower than the observed one (24.5% factual vs. 22.7% counterfactual). The initial reduction observed in the prevalence of high-risk drinkers in March 2020 (13% factual vs. 17% counterfactual) was followed by a rapid increase and in December 2023, the prevalence was significantly higher than expected (19.7% vs. 16.9%). The fruit and vegetable consumption worsened and the factual prevalence of five-a-day in December 2023 was significantly lower than counterfactual (6.6% vs. 9.0%). Physical inactivity behaviour, following an initial rise in March 2020 (albeit not statistically significant), appeared to decrease in December 2023, with the factual prevalence lower than counterfactual (26.9% vs. 32.4%). The results of this study remained robust in the sensitivity analysis for all of the four risk factors considered, where we tested April 2020 and May 2020 as “intervention” periods. All tested models showed no significant differences compared to those based on March 2020 (see Figs. 1, 2, 3 and 4 in Supplementary Materials).

## Discussion

This study used the surveillance system PASSI to examine the impact of COVID-19 pandemic outbreak on unhealthy habits of people residing in Italy, analysing how the prevalence of the four main behavioural indicators (tobacco, high-risk alcohol use, fruit and vegetables consumption and physical inactivity) changed after March 2020, the month in which lockdown restrictions were introduced with COVID-19 epidemiological emergency.

The results showed at December 2023 the achievement of a plateau for current smokers, a worsening in the high-risk alcohol consumption and in fruit and vegetable intakes while a reduction in physical inactivity.

As reported in a systematic review of 2022 [29], the effects of the pandemic on smoking behaviours are uncertain and appear to depend on the cigarette type: dual users and e-cigarette-only users with a high dependence on e-cigarettes showed increased consumption, while considering quit intentions, attempts and cessation success, the rates increased among cigarettes-only users more than e-cigarettes ones. It may be due to the fear of more severe symptoms and outcomes of COVID-19 among smokers [29]. Another systematic review showed a decrease in smoking consumption during the pandemic period. Worldwide global lockdowns and a lower social interaction may have limited the chances for social smoking and even restricted access to purchasing cigarettes [11]. Moreover, smoking may be viewed as an unhealthy mechanism for stress, with individuals feeling a lack of control over the quantity of cigarettes they consume and the found increase could be explained also by sensations of boredom during the lockdown [30, 31]. Our study suggests that in Italy the impact of COVID-19 on smoking behavior contributed to the achievement of a plateau contrasting with the gradual deceleration of the downward trend seen in the prevoius period. We could not exclude that these findings are also a consequence of the transition to novel and emerging tobacco and nicotine products (such as e-cigarette, Heat Tobacco Products (HTPs), nicotine pounches) which change the tradional concept of smoke. Moreover, after 10 years the introduction of e-cigarettes and 5 years after the launch of HTPs, the majority of adult smokers in Italy remained loyal to conventional tobacco cigarettes, with almost two out of three of novel product users continuing to smoke traditional cigarettes [32].

Our results highlighted the high-risk alcohol consumption has changed abruptly, with a drastic decline in the months following March 2020 until reached higher levels than the predicted, exceeding pre-pandemic levels, as seen toward the end of 2023. The reduction found in the months immediately after March 2020 is in line with literature. This result could be explained because lockdown measures impact both the locations where individuals can consume alcohol and their opportunities to socialize, actually many bars, pubs and such places remained closed; in addition, the ban that had been introduced prohibited going out especially in March and April 2020 [33, 34].

The prevention of diseases, such as diabetes, obesity, hypertension, and metabolic syndrome-related pathologies, in industrialized countries is significantly influenced by eating and lifestyle habits, along with the adoption of a well-balanced diet [35]. As shown by the literature, although the stringent measures may aid in interrupting the transmission pathway of the virus and flattening the infection curve, such restrictions could modify individual lifestyles as fruit and vegetable intake. Even though Italy is characterized by the Mediterranean diet, the intake of fruits and vegetables remained lower than the recommended levels set by dietary guidelines [36]. The decrease observed for this indicator in the post-pandemic period could be explained by the willingness to limit boredom due to interruption of the daily routine and outdoor activities and also alleviate fear and stress increasing consumption of high-calorie food and late-night eating, but also by the restricted access to regular grocery shopping led to a decline in the consumption of fresh foods, including fruits, vegetables [37]. Also, an Italian study found that improved dietary habits (higher consumption of fresh fruit, vegetables and fish) were strictly correlated with physical exercise and during the lockdown social isolation has impacted both aspects that are interrelated [38]. The period of lockdown induced shifts in consumer behavior, consequently impacting the quality of their diets. There was an inclination toward the consumption of processed foods, encompassing items like junk foods, snacks, ready-to-eat cereals, and convenience foods, accompanied by a decrease in the intake of healthy foods [33, 39].

It was well known that during the quarantine period, population was forced to remain indoors, going outside only for essential requirements in close proximity, thereby restricting social gatherings and physical activities. Certainly, this situation resulted in decreased physical activity levels and in increased physical inactivity time [35]. The increase found for physical inactive people in this study was in line with literature, only during the last month of 2022 was found a significative decreased physical activity reaching values similar to the pre-pandemic period. These results could be explained considering two different aspects: on the one hand, as described broadly for previous factors, lockdown restriction measures have had a strong impact, on the other hand for this specific indicator should not forget the massive use of smart working, until the onset of the pandemic, this work mode was not widespread in Italy. Jointly, the adoption of remote work, limited walking or use of public transport to work and reduced opportunities for practicing sports increased a physical inactivity lifestyle [40].

This study has some strengths and limitations. This is the first study in Italy that use a long time series, from 2008 to 2023, regarding behavioural risk factors for NCDs, based on representative samples of the adult population residing in Italy, that are be able to offer an ongoing perspective of reality of the impact of the COVID-19 pandemic on lifestyle. The robustness of the methodological framework on which PASSI is based, along with the opportunity offered to have access to a large and robust dataset covering over a decade of observation, represents an additional strengths of this study. Additionally, ITS is considered a robust quasi-experimental design for evaluating the longitudinal impact of an intervention, as it accounts for both random month-to-month fluctuations and the underlying trend in the analysis.

The results of our study were also reliable to sensitivity analyses in which were tested two other interventions period (April 2020 and May 2020) showing no significant differences compared to those based on March 2020.

The continuous data collection that characterizes PASSI makes it as a crucial tool for assessing trends, not only in the short term but also in the medium and long term, in behaviors and prognostic factors over time and it will enable future evaluations of the lasting effects of various events, including emergencies like the COVID-19 pandemic [41].

Limitations were mainly attributable to the self-reporting nature of data collection. Data cannot be validated with objective measures, except for sex and age, and collected information could be biased, generating underestimations or overestimations [42]. For example, smoking or alcohol consumption may be affected by bias for social desirability, and as for phisycal activity or fruits and vegatables assumption, recall bias could occur. In any way, self-reported data show robust consistency and sensitivity and they are valid information useful to monitor events over time [43], even because the PASSI interviewers are specially trained healthcare workers from the LHU Prevention Departments and used questionnaires did not change during the period under observation [17].

Another limitation arises from the absence of data from Lombardy for the years 2018–2023, as Lombardy has not been participating in the PASSI surveillance system since 2018. Unfortunately, the Lombardy region stands out as one of the primary areas where COVID-19 is prevalent in Italy.

More studies are needed to confirm the found results by continuing to monitor these behavioural risk factors over time, the analyzed time series could change trends by adding new observed points during the future, and the impact of COVID-19 may have been smaller or even stronger, or it may have been temporary or permanent.

Furthermore the results presented were valid for adults aged 18–69 residing in Italy, but each behavioural risk factor or lifestyle could be in deep analyzed stratifying by gender, age groups, social and economic status, to underline any particular trend or slope in subgroups. It is well known that smoking habits are more frequent in males or in socio-economic disadvantaged people or, on the contrary, alcohol consumption at high risk is associated more with young age or high social standing, it is important to understand if those sub-populations experienced the same impact by COVID-19 on those lifestyle than the whole population.

## Conclusion

The results of this study indicated a worsening trend in high-risk alcohol consumption and a decline in fruit and vegetable intake, while physical inactivity showed some improvement, at least by the end of 2023. Smoking behaviors appeared to achieve a plateau, in contrast to the gradual decline observed in previous years. These changes could have both short- and long-term impacts on people’s mental and physical health. Understanding shifts in these lifestyle indicators is crucial for the proper design of interventions aimed at reducing the burden of NCDs.

Moreover, this kind of results could assist public health policymakers in formulating strategies aimed at preventing the health repercussion, at short, medium, and long-term, arising from lifestyle changes due to the policy responses to COVID-19 or to potential future pandemics, taking into account not only needed containment and mitigation policies to flatten the peak of infection but also the impact on risk factor for NCDs, which still remain the leading cause of disability and mortality in Italy.

## Electronic supplementary material

Below is the link to the electronic supplementary material.

Supplementary Material 1

## Data Availability

The datasets analysed during the current study are not publicly available due to privacy restrictions but are available from the corresponding author on reasonable request.

## Abbreviations

AAPOR: American Association for Public Opinion Research
AIC: Akaike Information Criterion
ARMA: AutoRegressive Moving Average
AU: Alcoholic Units
CDC: Centers for Disease Prevention and Control
CI: Confidence Intervals
EU: European Union
GLS: Generalised Least Squares
ISS: Istituto Superiore di Sanità
ITS: Interrupted Time Series
LHU: Local Health Unit
NCDs: Non Communicable Diseases
PASSI: Progressi delle Aziende Sanitarie per la Salute in Italia
WHO: World Health Organization
