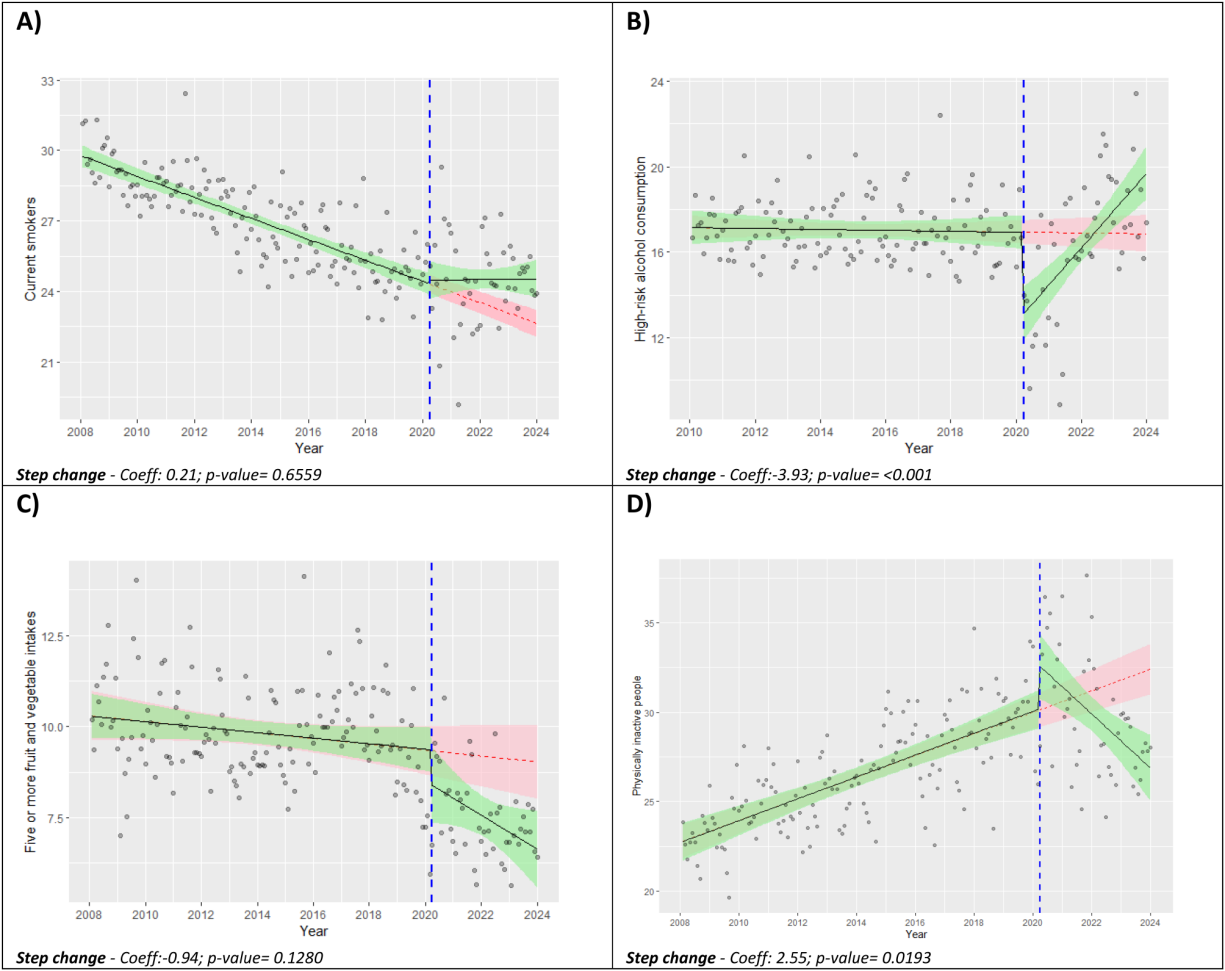# Publisher Correction to: Did the pandemic change lifestyle behaviours in Italy? An interrupted time series analysis on the four main NCDs behavioural risk factors from 2008 to 2023

**DOI:** 10.1186/s12889-025-22147-y

**Published:** 2025-04-07

**Authors:** Federica Asta, Valentina Minardi, Benedetta Contoli, Valentina Possenti, Virginia Casigliani, Maria Masocco

**Affiliations:** 1 National Centre for Disease Prevention and Health Promotion, Istituto Superiore di Sanità, Rome, Italy; 2 Department of Translational Research and New Technologies in Medicine and Surgery, University of Pisa, Pisa, Italy

**Correction to: BMC Public Health 25, 799 (2025).**

10.1186/s12889-025-22062-2

In the original publication of this article there was an error with Fig. 1. Figure 1 only contained panel D due to an error in the publication process. The incorrect and correct figure are shown in this correction article. The original article has been updated. The publisher apologizes for the inconvenience to the authors & readers.

**Incorrect Fig. 1**.

**Figure:**
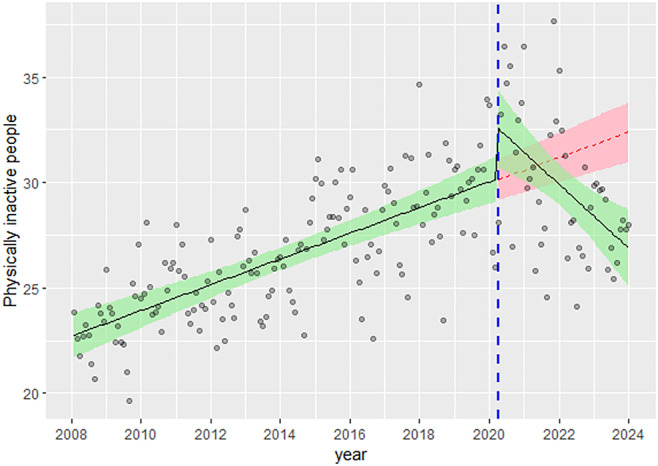

**Correct Fig. 1**.

**Figure:**