# Supplementary material for: Did the pandemic change lifestyle behaviours in Italy? An interrupted time series analysis on the four main NCDs behavioural risk factors from 2008 to 2023
Source: BMC Public Health. 2025 Feb 27;25:799. doi: 10.1186/s12889-025-22062-2 (PMC11869655; doi:10.1186/s12889-025-22062-2)
Supplement: Supplementary file 1 — Supplementary Material 1 [file 12889_2025_22062_MOESM1_ESM.docx]

**Supplementary Figure 1.** Interrupted time series models of monthly prevalence for current smokers shifting the intervention period at **April 2020** (**A**) and **May 2020** (**B**).

Counterfactual data (red line) and factual data (green line) with relative 95%IC, and weighted observed data (grey dots). PASSI 2008-2023.

| **A)**  **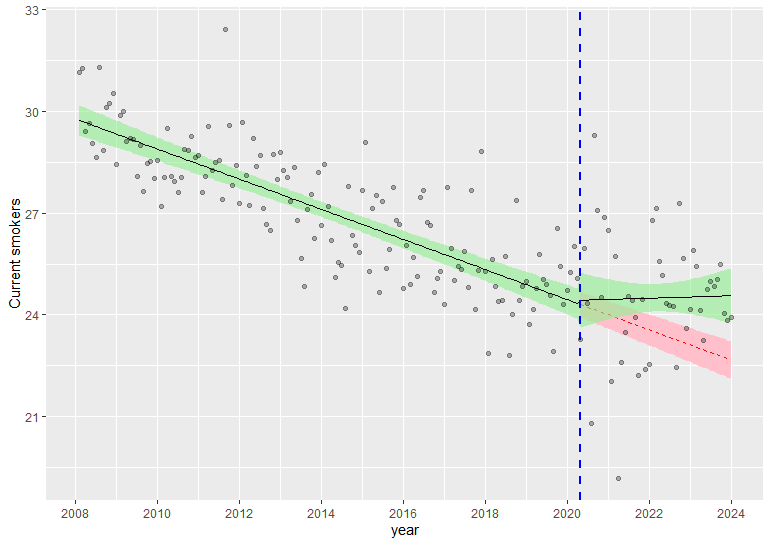**  ***Step change*** *- Coeff: 0.09; p-value= 0.8544* | **B)**  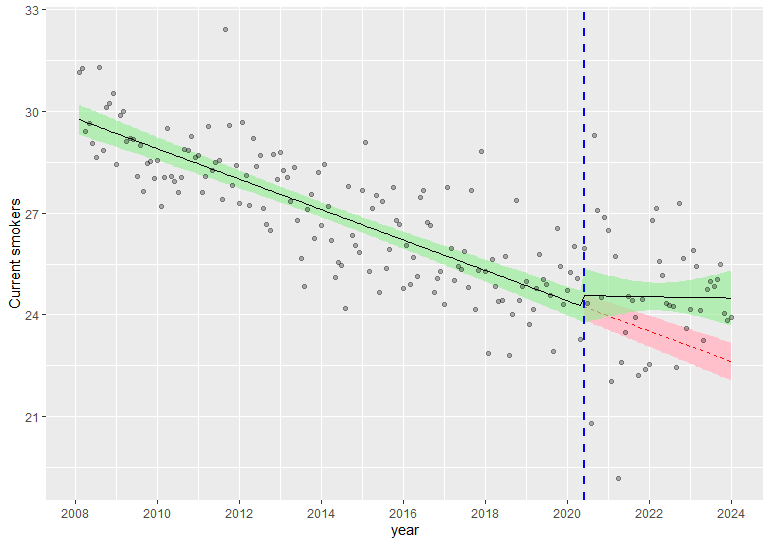  ***Step change*** *- Coeff: 0.30; p-value= 0.5233* |
| --- | --- |

**Supplementary Figure 2.** Interrupted time series models of monthly prevalence for people at high-risk alcohol use shifting the intervention period at **April 2020** (**A**) and **May 2020** (**B**).

Counterfactual data (red line) and factual data (green line) with relative 95%IC, and weighted observed data (grey dots). PASSI 2008-2023.

| **A)**  **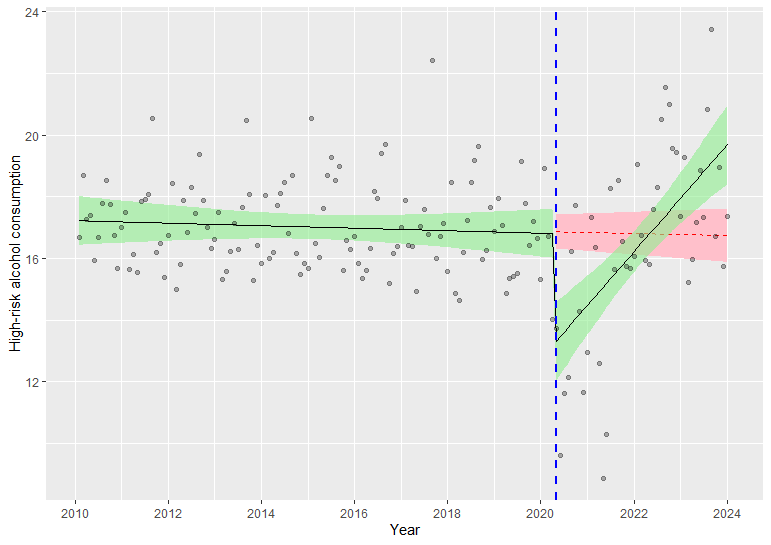**  ***Step change*** *- Coeff: -3.63; p-value<0.001* | **B)**  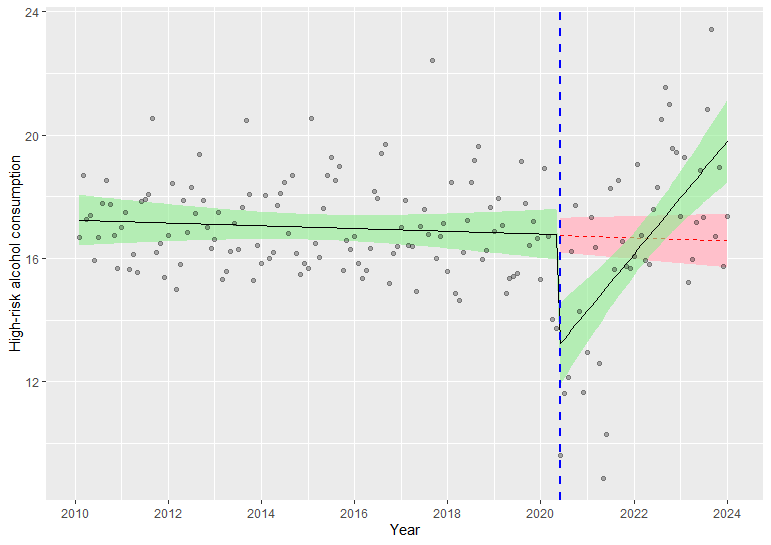  ***Step change*** *- Coeff: -3.69; p-value<0.001* |
| --- | --- |

**Supplementary Figure 3.** Interrupted time series models of monthly prevalence for people who eat 5 or more fruit and vegetable portions shifting the intervention period at **April 2020** (**A**) and **May 2020** (**B**).

Counterfactual data (red line) and factual data (green line) with relative 95%IC, and weighted observed data (grey dots). PASSI 2008-2023.

| **A)**  **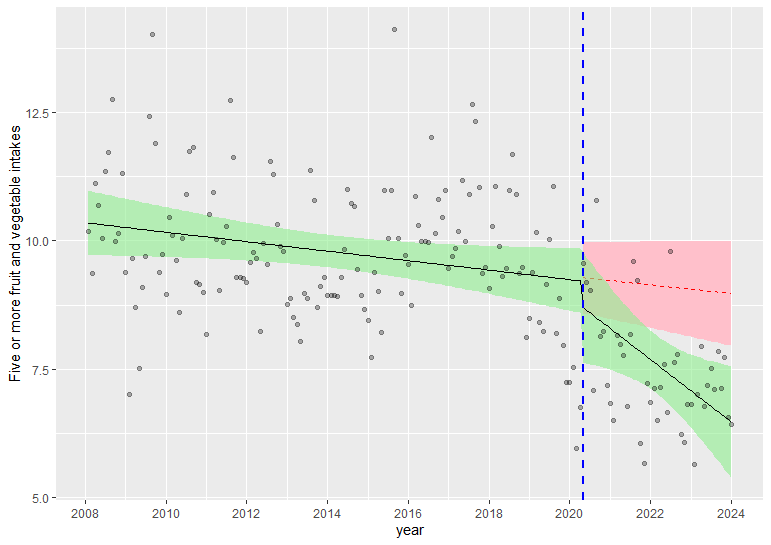**  ***Step change*** *- Coeff: -0.47; p-value=0.4542* | **B)**  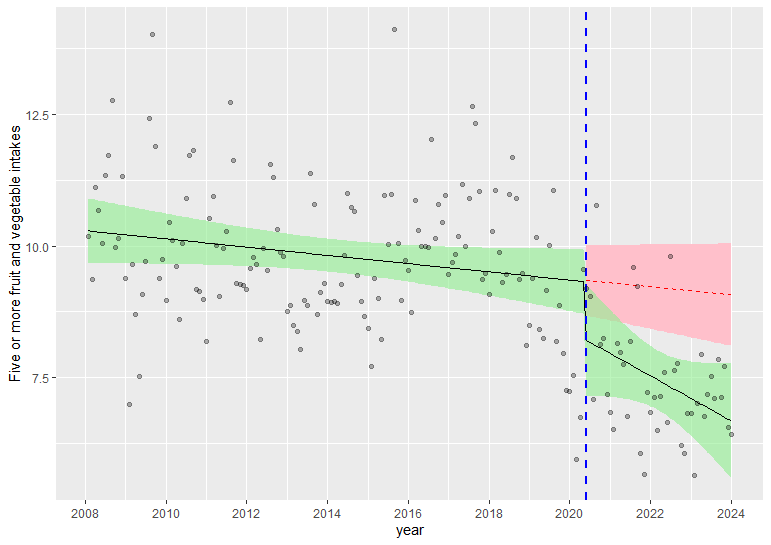  ***Step change*** *- Coeff: -1.08; p-value=0.0859* |
| --- | --- |

**Supplementary Figure 4.** Interrupted time series models of monthly prevalence for physical inactive people shifting the intervention period at **April 2020** (**A**) and **May 2020** (**B**).

Counterfactual data (red line) and factual data (green line) with relative 95%IC, and weighted observed data (grey dots). PASSI 2008-2023.

| **A)**  **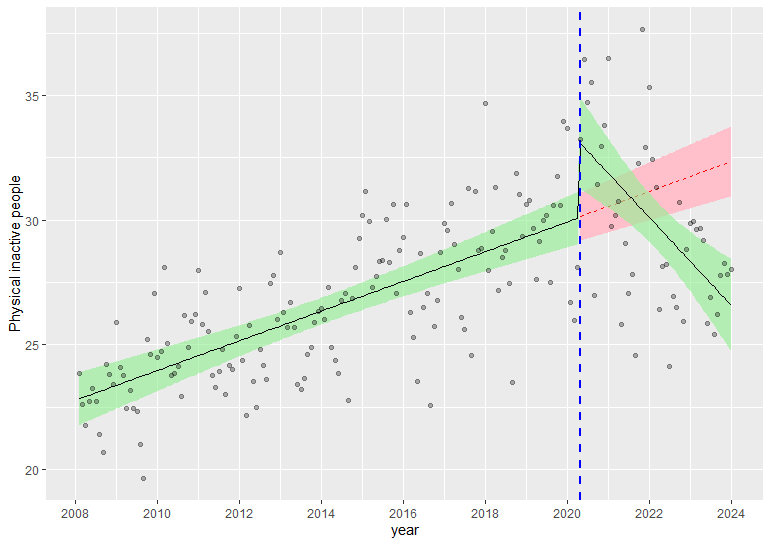**  ***Step change*** *- Coeff: 3.14; p-value=0.0043* | **B)**  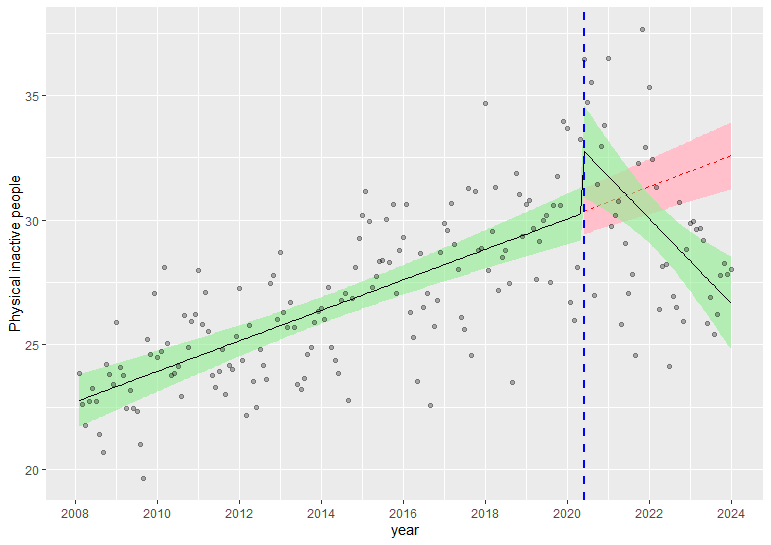  ***Step change*** *- Coeff: 2.65; p-value=0.0161* |
| --- | --- |
